# Association between controlling nutritional status (CONUT) score and amputation risk factors in T2DM patients with DFU

**DOI:** 10.3389/fendo.2026.1690617

**Published:** 2026-01-28

**Authors:** Jie Xiang, Weijia Huang, Wei Gao, Yuanhui Tu, Yongsheng Zhang

**Affiliations:** 1 Department of Clinical Nutrition, The First Affiliated Hospital of Guangxi Medical University, Nanning, China; 2 Department of Health Management, The First Affiliated Hospital of Guangxi Medical University, Nanning, China; 3 Department of Bone and Joint Surgery, The First Affiliated Hospital of Guangxi Medical University, Nanning, China

**Keywords:** amputation, CONUT score, DFU, risk factor, T2DM

## Abstract

**Background:**

Diabetic foot ulcer (DFU) is one of the serious complications of type 2 diabetes (T2DM). Malnutrition is associated with amputation in DFU patients. We aimed to use the CONUT score to investigate the risk factors associated with DFU amputation.

**Methods:**

A retrospective analysis was conducted on 387 DFU patients admitted to the First Affiliated Hospital of Guangxi Medical University from January 2024 to June 2025. The patients were divided into non-amputation group (n=231) and amputation group (n=156). Additionally, patients were divided into three groups based on the CONUT score. Demographic characteristics, blood biochemical indicators, amputation rate were measured. Multiple linear regression analysis, multivariate binary logistic regression analysis, subgroup analysis and receiver operating characteristic (ROC) curve analysis were conducted.

**Results:**

The overall amputation rate of DFU patients was 40.3%. The amputation group had longer hospital stays, larger foot ulcer areas, higher incidence of osteomyelitis and peripheral arterial disease (PAD) than those without amputation. In addition, the amputees had lower levels of albumin, prealbumin, hemoglobin and prognostic nutritional index, but higher levels of WBC, ESR, and CONUT score. As the CONUT score increased, the amputation rate of patients also increased. Logistic regression found that CONUT score, osteomyelitis and PAD were independent risk factors of amputation in DFU patients. Subgroups analysis showed CONUT score remained significantly associated with DFU amputation in the subgroups except in patients with HbA1c < 7%. ROC analysis showed that the AUC was 0.705 (95% CI: 0.652–0.758, p<0.001), indicating that CONUT score had good predictive performance for DFU amputation.

**Conclusion:**

CONUT score was associated with amputation in DFU patients. Early assessment of the patient’s nutritional status and improvement of malnutrition can reduce the risk of amputation.

## 1 Introduction

The aging population and the prevalence of obesity in China have led to a rapid increase in the population of T2DM. China has the largest population of diabetes in the world, with more than 118 million diabetes patients, of which more than 90% are type 2 diabetes (1). The prevalence of complications in patients with T2DM is also high. Diabetic foot ulcer (DFU) is one of the most common and serious complications in patients with diabetes. The global prevalence of DFU was 6.3%, while the prevalence rate in China was 5.7% (2). In addition, DFU significantly increased the risk of amputation. DFU patients often require hospitalization, with 15%-20% of patients undergoing lower limb amputation (3). The overall amputation rate of Chinese DFU patients was 19.03%, including 2.14% for major amputations and 16.88% for minor amputations (4). Amputation caused by DFU is the third most expensive complication of diabetes, and many patients are more afraid of amputation than death (5).

The increased nutritional and energy requirements for wound healing in DFU patients often lead to malnutrition. Malnutrition further delayed wound healing and reduced their quality of life significantly (6). Malnutrition is also associated with lower limb amputation in DFU (7). However, there is still limited research on the association between malnutrition and the risk of amputation in DFU. The Control of Nutritional Status (CONUT) score is a nutritional assessment according to objective examinations that is more suitable for clinical use due to its simplicity and ease of implementation (8). The purpose of this study is to explore the risk factors for amputation in T2DM patients with DFU, including CONUT score.

## 2 Patients and methods

### 2.1 Study participants

This study collected electronic medical records of 387 T2DM inpatients combined with DFU, including 231 non-amputation patients and 156 amputation patients. They were hospitalized in the department of bone and joint surgery or endocrinology, the First Affiliated Hospital of Guangxi Medical University from January 2024 to June 2025. Diagnosed T2DM and DFU according to the standards of the American Diabetes Association (9) and the World Health Organization (10). The inclusion criteria: (1) T2DM combined with DFU (Wagner grade 1-5) in the hospital; (2) adults aged over 18 years old. The following are exclusion criteria: (1) T1DM or other types of diabetes; (2) suffering from malignant tumors or severe vital organ failure; (3) foot ulcer caused by long-term use of glucocorticoid or other drugs.

### 2.2 Clinical data collection

The patient’s clinical data came from the electronic medical record system, including: (1) clinical characteristics including age, gender, BMI, T2DM duration, hospitalization days, foot ulcer area, smoking history, alcohol drinking history, handgrip strength, osteomyelitis, peripheral arterial disease (PAD); (2) diabetic complications and comorbidities including diabetic retinopathy, diabetic nephropathy, diabetic peripheral neuropathy, hypertension, coronary heart disease; (3) laboratory results including uric acid, albumin, prealbumin, WBC, HbA1c, neutrophils, lymphocyte, hemoglobin, serum creatinine, blood urea nitrogen, hs-CRP, ESR, fibrinogen, 25-OH-VD, TC, TG, HDL-C, LDL-C; (4) nutritional status assessment including Controlling Nutritional Status (CONUT) score and Prognostic Nutritional Index (PNI). Assess the severity of DFU by Wagner grading 1–5 levels.

### 2.3 Amputation related definitions

Amputation was usually divided into major amputation (above the ankle joint level) and minor amputation (below the ankle joint level, including toe amputation) (11).

### 2.4 Handgrip Strength

Handgrip strength is a simple method for assessing nutritional status and muscle strength, which is feasible in clinical practice (12). An electronic grip strength meter (CAMRY EH101, Xiangshan, China) was used to assess handgrip strength. Refer to the consensus of Asian Sarcopenia Working Group on the diagnosis and treatment of sarcopenia in 2019, when the male grip strength was less than 28kg or the female grip strength was less than 18kg, it was defined as low grip strength (13).

### 2.5 CONUT score

The score range for Control of Nutritional Status (CONUT) was 0 to 12, with higher scores representing poorer nutritional status, 0–1 score meaning normal nutritional status, 2–4 score meaning mild malnutrition, 5–8 score meaning moderate malnutrition, 9–12 score meaning to severe malnutrition (**Table 1**).

**Table 1:** CONUT score.

Parameter	Normal	Mild	Moderate	Severe
Albumin(g/dl)	3.5-4.5	3.0-3.49	2.5-2.9	<2.5
score	0	2	4	6
Lymphocytes(/mm^3^)	>1600	1200-1599	800-1199	<800
score	0	1	2	3
Cholestrol(mg/dl)	>180	140-180	100-139	<100
score	0	1	2	3
Total score	0-1	2-4	5-8	9-12
Malnutrition degree	Normal	Mild	Moderate	Severe

CONUT score, Control of Nutritional Status score.

### 2.6 PNI

The prognostic nutritional index (PNI) calculation formula: serum albumin (g/L) + 5 × total lymphocyte count (10^9^/L) (14). PNI According to the PNI score, >50 indicates normal nutritional status, 45–50 indicates mild malnutrition, 40–45 indicates significant malnutrition, and<40 indicates severe malnutrition, respectively (15).

### 2.7 Statistical analyses

SPSS 23.0 software, Graphpad prism 10.0 software and Zstats platform (www.medsta.cn/software) were used for statistical analysis. The continuous variables in this study were non-normally distributed detected by Shapiro-Wilk test, which described as median and interquartile range (IQR, 25-75%) and compared by Mann-Whitney test. Categorical variables were presented in percentage (%) and compared using Chi-square test. Kruskal-Wallis test was used for non-parametric testing of multiple independent samples. Multiple linear regression analysis selected variables with statistical significance for logistic regression analysis. The risk factors of DFU amputation were analyzed using multivariate binary logistic regression analysis and conducting subgroup analysis to further validate the stability of the model. Receiver Operating Characteristic (ROC) curve analysis was constructed to evaluate the discriminative performance of amputation in DFU patients. P<0.05 was statistically significant.

## 3 Results

### 3.1 Clinical Characteristics in non-amputation and amputation groups

387 T2DM with DFU patients (290 Males and 97 females) were retrospectively analysed. The patients were divided into non-amputation group (n=231) and amputation group (n=156), with an amputation rate of 40.3%. The sample size of patients with major amputations was relatively small, so they were not included in this study. Therefore, all patients in the amputation group had minor amputations. **Table 2** showed that there were no significant differences in age, gender, BMI, T2DM duration, drinking history, diabetic retinopathy, diabetic nephropathy, diabetic peripheral neuropathy, hypertension, coronary heart disease and low handgrip strength between the two groups (p>0.05). However, in contrast to the non-amputation group, amputation group had longer hospitalization days [15 (14, 17) vs 8 (7, 10), p<0.001] and larger foot ulcer areas [35 (20, 50) vs 6 (4, 15), p<0.001]. The amputated patients also had a higher smoking proportion (43.6% vs 31.2%, p = 0.013), prevalence of osteomyelitis (34.0% vs 7.4%, p<0.001) and PAD (82.1% vs 26.4%, p<0.001) than those without amputation.

**Table 2:** Demographic and clinical characteristics.

Variable	Non-amputation (n=231)	Amputation (n=156)	p-value
Age (years)	61 (59, 71)	61 (57, 68)	0.122
BMI (kg/m^2^)	22.3 (20.7, 24.2)	22.3 (20.0, 24.7)	0.848
Male (%)	176 (76.2%)	114 (73.1%)	0.488
T2DM Duration (years)	9 (6, 14)	8 (6, 12)	0.130
Hospitalization days	8 (7, 10)	15 (14, 17)	<0.001
Foot ulcer area (cm^2^)	6 (4, 15)	35 (20, 50)	<0.001
Smoking history	72 (31.2%)	68 (43.6%)	0.013
Drinking history	57 (24.7%)	46 (29.5%)	0.293
Diabetic retinopathy	75 (32.5%)	54 (34.6%)	0.660
Diabetic nephropathy	71 (30.7%)	54 (34.6%)	0.423
DPN	188 (81.4%)	129 (82.7%)	0.743
Hypertension	143 (61.9%)	104 (66.7%)	0.339
Coronary heart disease	54 (23.4%)	43 (27.6%)	0.351
Low handgrip strength	107 (46.3%)	83 (53.2%)	0.184
osteomyelitis	17 (7.4%)	53 (34.0%)	<0.001
PAD	61 (26.4%)	128 (82.1%)	<0.001

BMI, body mass index; DPN, diabetic peripheral neuropathy; PAD, peripheral arterial disease.

### 3.2 Laboratory results

There were no significant differences in HbA1c, Scr, BUN, Uric acid, Lymphocyte, hs-CRP, 25-OH-VD, HDL-C (p>0.05) between the two groups. Compared with the non-amputees, amputation patients had lower levels of nutritional indicators including albumin [34.2 (31.3, 37.7) vs 38.0 (35.1, 38.9), p<0.001], prealbumin [188.0 (154.9, 220.5) vs 199.3 (166.6, 241.8), p = 0.014], hemoglobin [102.5 (89.0, 129.0) vs 118.0 (97.0, 130.0), p = 0.001], TC [3.35 (2.74, 4.10) vs 3.74 (3.44, 4.20), p<0.001], TG [0.64 (0.57, 0.88) vs 1.20 (0.81, 1.42), p<0.001], LDL-C [1.68 (1.58, 2.10) vs 1.78 (1.57, 2.52), p = 0.001], and PNI [42.0 (38.5, 45.6) vs 44.6 (42.5, 47.3), p<0.001], but higher levels of WBC, neutrophils, ESR, Fibrinogen and CONUT score (p<0.001, **Table 3**).

**Table 3:** Laboratory results.

Variable	Non-amputation (n=231)	Amputation (n=156)	p-value
HbA1c (%)	8.4 (7.3, 9.8)	8.9 (7.3, 10.4)	0.147
Scr (umol/l)	87.0 (60.5, 123.0)	84.9 (58.0, 108.2)	0.177
BUN (mmol/l)	7.2 (6.0, 9.2)	7.2 (6.0, 9.0)	0.553
Uric acid (umol/l)	283.0 (233.0, 375.6)	282.7 (233.0, 362.2)	0.291
Albumin (g/l)	38.0 (35.1, 38.9)	34.2 (31.3, 37.7)	<0.001
Prealbumin (mg/l)	199.3 (166.6, 241.8)	188.0 (154.9, 220.5)	0.014
White blood cell (×10^9^/l)	8.4 (7.2, 9.5)	8.8 (7.5, 11.5)	<0.001
Neutrophils (×10^9^/l)	5.5 (4.9, 6.7)	6.7 (4.3, 8.4)	<0.001
Lymphocyte (×10^9^/l)	1.5 (1.4, 1.7)	1.6 (1.5, 1.7)	0.150
Hemoglobin (g/l)	118.0 (97.0, 130.0)	102.5 (89.0, 129.0)	0.001
hs-CRP (mg/l)	5.0 (3.6, 8.9)	6.0 (2.4, 11.0)	0.185
ESR (mm/h)	51.0 (29.8, 54.5)	71.7 (54.1, 110.9)	<0.001
Fibrinogen (g/l)	4.2 (4.0, 4.6)	4.9 (4.3, 5.5)	<0.001
25-OH-VD (nmol/l)	31.9 (25.1, 47.3)	30.1 (25.9, 35.7)	0.060
Total cholesterol (mmol/l)	3.74 (3.44, 4.20)	3.35 (2.74, 4.10)	<0.001
Triglyceride (mmol/l)	1.20 (0.81, 1.42)	0.64 (0.57, 0.88)	<0.001
HDL-C (mmol/l)	1.10 (0.81, 1.47)	1.10 (0.94, 1.48)	0.253
LDL-C (mmol/l)	1.78 (1.57, 2.52)	1.68(1.58, 2.10)	0.001
Prognostic Nutritional Index	44.6 (42.5, 47.3)	42.0 (38.5, 45.6)	<0.001
CONUT score	2 (1, 3)	4 (2, 5)	<0.001

HbA1c, glycosylated hemoglobin; Scr, serum creatinine; BUN, blood urea nitrogen; hs-CRP, hypersensitive C-reactive protein; ESR, erythrocyte sedimentation rate; HDL-C, high density lipoprotein cholesterol; LDL-C, low density lipoprotein choleste; CONUT score, Controlling Nutritional Status score.

### 3.3 Amputation rates and clinical characteristics of DFU patients with different CONUT scores

387 DFU patients were grouped according to CONUT score as follows: 93 cases in the normal nutrition group, 212 cases in the mild malnutrition group, 82 cases in the moderate malnutrition group, and 3 cases in the severe malnutrition group, respectively. Due to the small number of patients with severe malnutrition (3 cases), moderate malnutrition and severe malnutrition were combined into one group. The amputation rates of DFU patients with normal nutrition, mild malnutrition, and moderate to severe malnutrition were 15.1%, 40.6%, and 68.3%, respectively. As the CONUT score increased, the amputation rate, hospitalization days, foot ulcer area and low handgrip strength of DFU patients also increased. There were no significant differences in Age, BMI, T2DM duration and WBC (p > 0.05) between the three groups. However, there were statistically significant differences in HbA1c, hemoglobin, albumin, 25-OH-VD, TC, TG, HDL-C, LDL-C (p < 0.05) among DFU patients with different CONUT scores. The PNI index of the moderate to severe malnutrition group (CONUT 5–12 score) was also the lowest, indicating consistency between these two nutritional assessment tools for malnutrition (**Table 4**).

**Table 4:** Clinical characteristics of DFU patients with different CONUT scores.

Variable	0-1 (n=93)	2-4 (n=212)	5-12 (n=82)	p-value
Age (years)	61 (59, 65)	62 (58,71)	63 (57, 71)	0.170
BMI (kg/m^2^)	22.5 (20.9, 24.0)	22.4 (19.9, 25.0)	22.0 (19.5, 24.2)	0.437
Amputation (%)	14 (15.1%)	86 (40.6%)	56 (68.3%)	<0.001
Hospitalization days	8 (8, 11)	12 (8, 15)	14 (10, 16)	<0.001
Foot ulcer area (cm^2^)	6 (4, 20)	20 (6, 30)	20 (12, 50)	<0.001
T2DM duration (years)	8 (6, 14)	9 (6, 12)	10 (6, 13)	0.525
Low grip strength (%)	14 (15.1%)	118 (55.7%)	54 (65.9%)	<0.001
HbA1c (%)	9.5 (7.5, 11.6)	8.3 (7.3, 9.5)	8.2 (7.3, 9.8)	0.003
WBC (×10^9^/l)	8.4 (7.2, 9.9)	8.4 (7.2, 9.9)	8.4 (7.3, 10.4)	0.964
Hemoglobin (g/l)	123 (98, 134)	109 (93, 130)	104 (90, 125)	0.001
Albumin (g/l)	38.4 (37.8, 39.0)	36.4 (33.4, 38.1)	30.7 (26.8, 33.3)	<0.001
25-OH-VD (nmol/l)	32.8 (24.1, 43.9)	31.1 (25.9, 39.6)	26.6 (23.4, 32.7)	0.001
TC (mmol/l)	4.13 (3.77, 5.05)	3.62 (3.24, 4.10)	3.28 (2.73, 3.62)	<0.001
TG (mmol/l)	0.90 (0.80, 1.28)	0.87 (0.57, 1.28)	0.64 (0.57, 1.28)	0.008
HDL (mmol/l)	1.10 (1.07, 1.47)	1.20 (0.90, 1.48)	1.01 (0.90, 1.20)	0.001
LDL (mmol/l)	2.50 (1.76, 3.17)	1.66 (1.57, 2.28)	1.66 (1.33, 2.19)	<0.001
PNI	47.4 (46.3, 49.4)	43.8 (41.9, 45.8)	37.5 (33.9, 40.3)	<0.001

BMI, body mass index; HbA1c, glycosylated hemoglobin; WBC, white blood cell; TC, Total cholesterol; TG, Triglyceride; HDL, High-Density Lipoprotein; LDL, Low-Density Lipoprotein; PNI, Prognostic Nutritional Index.

### 3.4 Multivariate binary logistic regression analysis of amputation risk factors in DFU patients

Pearson correlation analysis on variables such as age, BMI, Hospitalization days and CONUT was conducted, then included variables with p<0.05 in the multiple linear regression analysis. The multiple linear regression analysis results (**Table 5**) showed that ALB and TC had statistical significance (p<0.05) and included in the subsequent logistic regression analysis. DFU amputation was used as the dependent variable, while ALB, TC, CONUT score, smoking history, osteomyelitis and PAD were used as independent variables in the forward conditional logistic regression. The results showed that CONUT score (OR = 1.655, 95% CI: 1.423-1.926, p < 0.001), osteomyelitis (OR = 3.817, 95% CI: 1.905-7.647, p < 0.001), PAD (OR = 12.602, 95% CI: 6.955-22.836, p < 0.001), were independent risk factors of amputation in DFU patients (**Table 6**).

**Table 5:** Multiple linear regression analysis selects independent variables for logistic regression.

Model	B	SE	Beta	t	p-value	Tolerance	VIF
Hospitalization days	0.027	0.017	0.056	1.590	0.113	0.626	1.597
Foot ulcer area	-0.001	0.003	-0.014	-0.403	0.687	0.625	1.601
HbA1c	-0.034	0.025	-0.040	-1.362	0.174	0.901	1.109
Hb	0.001	0.003	0.015	0.497	0.620	0.833	1.201
ESR	0.001	0.002	0.025	0.798	0.425	0.803	1.245
ALB	-0.311	0.015	-0.685	-21.026	0.000	0.724	1.382
TC	-0.755	0.083	-0.345	-9.073	0.000	0.532	1.879
TG	-0.401	0.090	-0.014	-0.451	0.652	0.781	1.280
HDL	0.028	0.217	0.004	0.127	0.899	0.809	1.237
LDL	0.051	0.096	0.019	0.534	0.594	0.593	1.687
Prealbumin	0.000	0.001	-0.013	-0.404	0.687	0.771	1.297
Neutrophils	-0.013	0.020	-0.019	-0.641	0.522	0.886	1.129
Fibrinogen	0.094	0.098	0.029	0.956	0.340	0.822	1.216

SE, standard error; VIF, variance inflation factor; HbA1c, glycosylated hemoglobin; Hb, hemoglobin; ESR, erythrocyte sedimentation rate; TC, Total cholesterol; TG, Triglyceride; HDL, High-Density Lipoprotein; LDL, Low-Density Lipoprotein.

**Table 6:** Multivariate binary logistic regression analysis of amputation risk factors in DFU patients.

Variable	OR	95%CI	p-value
CONUT score	1.655	1.423-1.926	<0.001
Osteomyelitis	3.817	1.905-7.647	<0.001
PAD	12.602	6.955-22.836	<0.001

TC, Total cholesterol; CONUT score, Controlling Nutritional Status score.

We further assessed the effect of the CONUT score in the subgroups of DFU patients (**Table 7**). The results showed an increase in CONUT score was still significantly associated with DFU amputation in the subgroups (p < 0.05). However, in patients with HbA1c < 7%, this association was not significant (p = 0.085).

**Table 7:** Subgroup analysis of the association between CONUT and DFU amputations.

Variables	n (%)	OR (95%CI)	p-value	p for interaction
All patients	387 (100.00)	1.49 (1.32-1.68)	<0.001	
Gender				0.187
Male	290 (74.94)	1.44 (1.27-1.65)	<0.001	
Female	97 (25.06)	1.79 (1.32-2.41)	<0.001	
Age				0.157
<60 years	136 (35.14)	1.34 (1.13-1.60)	0.001	
≥60 years	251 (64.86)	1.60 (1.36-1.89)	<0.001	
BMI				0.053
<24 kg/m^2^	279 (72.09)	1.40 (1.23-1.60)	<0.001	
≥24 kg/m^2^	108 (27.91)	1.88 (1.41-2.51)	<0.001	
HbA1c				0.123
<7%	64 (16.54)	1.24 (0.97-1.59)	0.085	
≥7%	323 (83.46)	1.56 (1.36-1.79)	<0.001	
T2DM duration				0.404
<10 years	210 (54.26)	1.60 (1.33-1.91)	<0.001	
≥10 years	177 (45.74)	1.44 (1.21-1.70)	<0.001	
Smoking				0.120
No	247 (63.82)	1.60 (1.35-1.88)	<0.001	
Yes	140 (36.18)	1.31 (1.10-1.57)	0.003	
Drinking				0.810
No	284 (73.39)	1.51 (1.30-1.75)	<0.001	
Yes	103 (26.61)	1.46 (1.16-1.84)	0.001	
Hypertension				0.446
No	140 (36.18)	1.41 (1.17-1.71)	<0.001	
Yes	247 (63.82)	1.56 (1.33-1.83)	<0.001	
CHD				0.414
No	289 (74.68)	1.45 (1.27-1.66)	<0.001	
Yes	98 (25.32)	1.64 (1.26-2.13)	<0.001	

OR, Odds Ratio; CI, Confidence Interval; BMI, body mass index; HbA1c, glycosylated hemoglobin; T2DM, type 2 diabetes; CHD, coronary heart disease.

### 3.5 ROC curve analysis evaluates the predictive performance of CONUT score for DFU amputation

**Figure 1:**
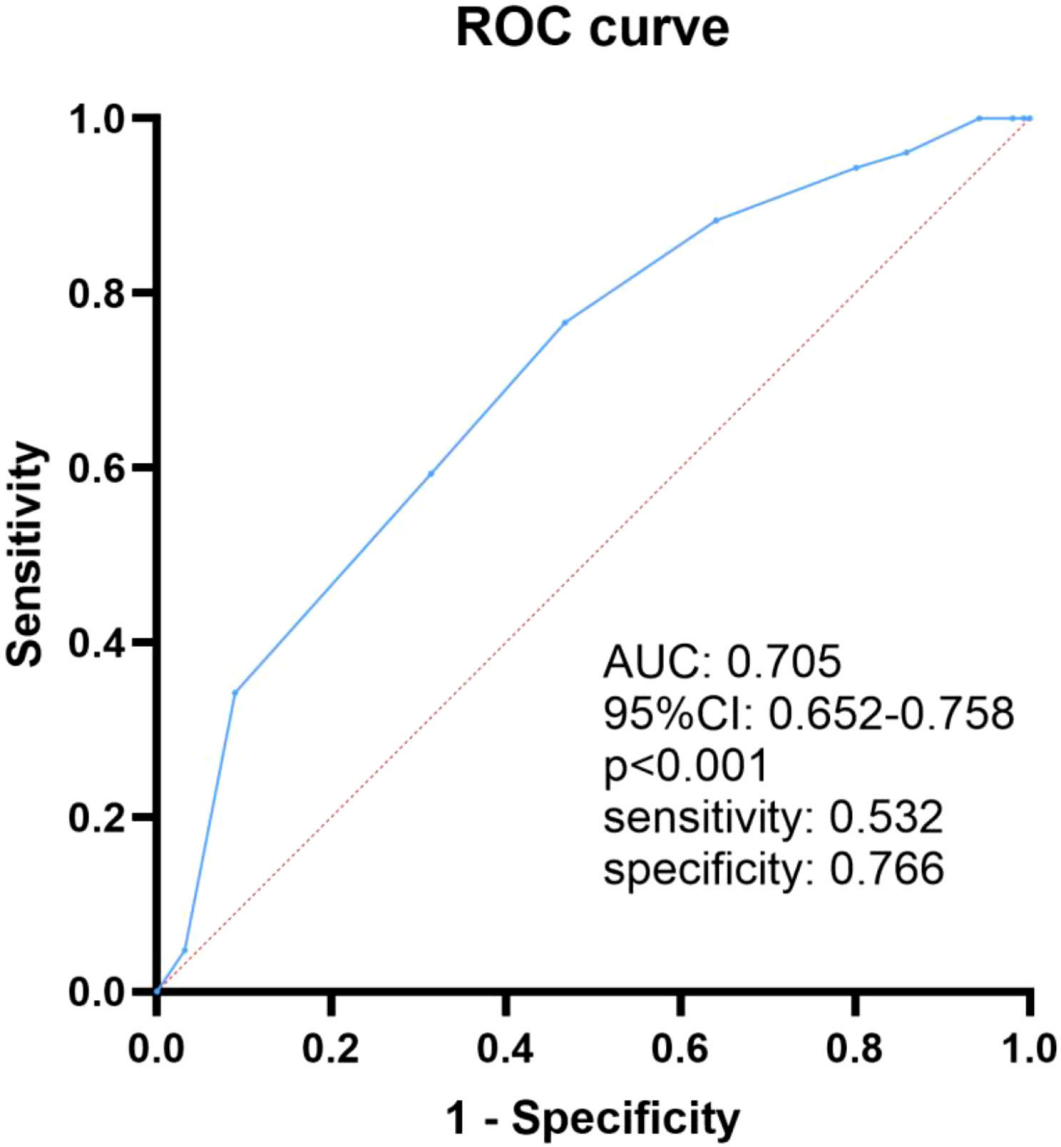
The ROC curve analysis of CONUT in predicting DFU amputation in T2DM patients.

We next draw an ROC curve to explore the predictive value of CONUT score for DFU amputation in T2DM patients. As shown in **Figure 1**, the area under the ROC curve was 0.705 (95% CI: 0.652–0.758, p<0.001). The optimal cutoff value of CONUT score was 3.5, corresponding to a Yoden index of 0.298, with sensitivity of 0.532 and specificity of 0.766.

## 4 Discussion

DFU is a global medical issue related to many risk factors, such as infection, peripheral arterial disease, and peripheral neuropathy. DFU is the main cause of nontraumatic amputation worldwidely, and nearly 85% patients with diabetes receiving nontraumatic lower limb amputation are caused by DFU (16). In China, about 4 million new DFU patients were diagnosed each year, with one DFU amputation occurring approximately every 30 seconds, making up 68% of the non-traumatic amputees (17). The wound healing of DFU is difficult, and about half of DFU patients experience lower limb amputation (18). In our study, the total amputation rate of DFU patients was 40.3%. Previous literature had shown that the amputation rate of Chinese DFU patients was 19.03%. The relatively high amputation rate reported in our study may be due to many critically ill patients coming to our hospital, including patients with DFU. In addition, patients did not pay attention at the initial stage of disease development, which led to the aggravation of DFU. The number of patients with major amputations was very small, significantly lower than that of China’s 2010 multi center diabetes major amputation survey (19), so they were not included in this study. The reduction of major amputation rate was related to the multidisciplinary cooperation in the therapy of DFU in our hospital, including the Department of Endocrinology, Clinical Nutrition, Bone and Joint Surgery, Rehabilitation. In this study, we found that the amputation group had longer hospitalization days, larger foot ulcer areas, and more frequent smoking than non-amputation group. Clinically, when the foot ulcer area of patients with DFU was larger and the infection was more serious, the risk of amputation increased significantly. Similarly, the FIELD study indicated that the smoking frequency of amputees was higher than that of non-amputees (20). Lin et al. found that smoking was a risk factor for amputation in DFU patients (21). Smoking can also lead to PAD in DFU patients (22).

We also found that the amputated patients had a higher incidence of PAD than those without amputation. PAD was a risk factor for amputation in DFU patients (23). Peripheral arterial ultrasound is a practical method for evaluating vascular diseases. In this study, patients with DFU were examined by lower limb ultrasound to determine whether they had PAD. PAD increased the risk of bacterial resistance and amputation for DFU patients (24). In our study, PAD remarkably increased risk of amputation. The Wagner classification of DFU was used to assess the severity of ulcers such as ulcer depth, osteomyelitis and gangrene. The severity of DFU ulcers usually increased with Wagner grading, and in particularly severe cases, amputation may be necessary. A systematic review related to DFU (25) emphasized the importance of Wagner grading in predicting amputation.

Foot infection is a common and serious problem in DFU patients. The infections usually started from open wounds on the skin, then spread to the underlying bones. Therefore, osteomyelitis is usually the result of long-term DFU, accompanied by peripheral artery disease, peripheral neuropathy and poor compliance of foot care (26). Osteomyelitis can increase the risk of amputation in DFU. In our study, the amputated patients had a higher incidence of osteomyelitis, infection inflammatory markers (white blood cell, neutrophil, ESR) than non-amputees. Logistic regression results also suggest that osteomyelitis was an independent risk factor for amputation in DFU patients. This indicated that the risk of amputation in DFU was related to the severity of wound infection, which was consistent with previous literature on risk factors for amputation in DFU (27).

Appropriate nutritional status is important for DFU patients. Malnutrition is very common in DFU patients (28). Due to the metabolic cost of repairing damaged tissue and the nutritional loss caused by wound inflammation exudate, DFU wounds had a negative impact on nutritional status (29). Micronutrient deficiencies were also common in DFU patients, which increased the risk of amputation (30). Studies found that Albumin and hemoglobin levels were important risk factors for major amputations in DFU patients (31, 32). Vitamin D insufficiency was associated with oxidative stress, and wound healing. Tang et al. revealed that Chinese T2DM patients with DFU had lower vitamin D levels (33). Vitamin D deficiency increased the risk of amputation in veterans with peripheral arterial disease (34). In our study, the amputation patients had lower levels of nutritional related indicators including albumin, prealbumin, hemoglobin, TC, TG, and LDL-C than the non-amputees. Low prognostic nutritional index (PNI) was related to higher amputation rate in DFU (35). In the PNI calculation formula, low serum albumin levels or low lymphocyte counts lead to low PNI scores, representing malnutrition. In this study, compared with the non-amputees, amputees had lower levels of albumin, resulting in lower PNI.

DFU patients need to undergo timely nutritional assessment. There are currently many nutritional assessment methods in clinical practice, such as nutritional risk screening 2002, Subjective Global Assessment, Mini Nutritional Assessment, all of which are obtained through methods such as inquiring about medical history, physical examination, and patient self-assessment. These methods are subjective and may affect the accuracy of the evaluation results. CONUT score is a nutritional assessment tool based on objective examination that uses Lymphocytes, Cholestrol, and serum ALB levels to calculate immune defense function, calorie burning ability, and protein reserve ability, thereby accurately and objectively evaluating the patient’s nutritional status (36). The CONUT score was associated with ulcer healing in patients with critical limb ischemia (37). In this study, patients were divided into three groups including normal nutrition, mild malnutrition, and moderate to severe malnutrition based on the CONUT score. The amputation rates of DFU patients with normal nutrition, mild malnutrition, and moderate to severe malnutrition were 15.1%, 40.6%, and 68.3%, respectively. As the CONUT score increased, the amputation rate of DFU patients also increased significantly. Logistic regression analysis found that the risk of amputation increased approximately 1.7 times with each unit increase in the CONUT score. Handgrip strength was often used as a marker of muscle strength, and was also one of the tests for evaluating malnutrition and sarcopenia (38). Low handgrip strength was related to DFU in T2DM geriatric patients (39). In our study, compared with the other two groups, the moderate to severe malnutrition group had more frequent occurrences of low grip strength. Moreover, the moderate to severe malnutrition group had longer hospital stays, larger foot ulcer areas, and lower nutritional indicators than the other two groups. Therefore, the CONUT score can be used for nutritional evaluation of DFU patients and predict the risk of amputation.

There are still some limitations in our study. Firstly, it was a retrospective study and lacks indicators such as body composition analysis to comprehensively evaluate the nutritional status of DFU patients. Secondly, it was a single center study that only included Chinese adults from the First Affiliated Hospital of Guangxi Medical University. The relatively high amputation rate reported in our study may be due to the high proportion of hospitalized patients with severe DFU. However, these samples cannot represent the overall DFU situation and may have selection bias. Thirdly, due to the small number of patients with major amputations, the causes and risk factors for major amputations and minor amputations may be different. Combining these two different outcomes under one “amputation” heading may result in the loss of important clinical information. Therefore, patients with major amputations were not included in this study. More researches including large sample, multi-center studies are needed in the future.

## 5 Conclusion

This study found that CONUT score, osteomyelitis and PAD were independent risk factors for amputation in T2DM patients with DFU. The CONUT score can be used for nutritional evaluation of DFU patients and predict the risk of amputation. Early assessment of the patient’s nutritional status and improvement of malnutrition may reduce the risk of amputation.

## Data Availability

The original contributions presented in the study are included in the article/supplementary material. Further inquiries can be directed to the corresponding author.
